# Genetic diversity and different cross-neutralization capability of porcine circovirus type 2 isolates recently circulating in South Korea

**DOI:** 10.1186/s12917-020-02549-3

**Published:** 2020-09-14

**Authors:** Seok-Jin Kang, Hyeonjeong Kang, Su-Hwa You, Hye Jeong Lee, Nakhyung Lee, Bang-Hun Hyun, Sang-Ho Cha

**Affiliations:** 1 Viral Disease Division, Animal and Plant Quarantine Agency, 177, Hyeoksin 8-ro, Gimcheon-si, Gyeongsangbuk-do 39660 Republic of Korea; 2 KBNP, 415, Heungan-daero, Dongan-Gu, Anyang-si, Gyeonggi-do 14059 Republic of Korea

**Keywords:** Porcine circovirus type 2, Genotype, Antigenic diversity, Neutralization

## Abstract

**Background:**

Porcine circovirus type 2 (PCV2) is a small single-stranded DNA virus and a primary cause of PCV-associated diseases (PCVAD) that result insubstantial economic loss for swine farms. Between 2016 and 2018, PCV2 field viruses were isolated from PCVAD-affected swine farms in South Korea and investigated for genetic and antigenic heterogeneity.

**Results:**

The genetic analysis of ORF2 showed that the genotype of the Korean PCV2 field isolates has been rapidly shifted from PCV2a or 2b to mutant PCV2b known as PCV2d with 82.6 to 100% amino acid sequence similarity. PCV2-specific monoclonal antibodies (mAbs) demonstrated variable antigen-binding activity to four representative Korean PCV2 field isolates [QIA215 (PCV2a), QIA418 (PCV2b), QIA169 (PCV2d), and QIA244 (PCV2d)] without genotype specificity, and one mAb showed neutralization activity to QIA215. In a cross-virus neutralization assay using anti-PCV2 sera of pigs and guinea pigs injected with a commercial vaccine and the Korean PCV2 field isolates, the anti-porcine sera of a commercial vaccine had high neutralization activity against QIA215 and QIA418 with statistically lower activity against PCV2d viruses. Anti-guinea pig sera of QIA215, QIA418, QIA169, and a commercial vaccine had high neutralization activity against all of the viruses with significantly lower activity against QIA244. Importantly, anti-guinea pig sera of QIA244 had high neutralization activity against all of the viruses.

**Conclusions:**

This study confirmed genetic and antigenic diversity among recent PCV2 field isolates in Korean swine farms, and the strain-based difference in virus neutralization capability should be considered for more effective control by vaccination.

## Background

Porcine circovirus type 2 (PCV2) causes severe economic loss in the swine industry worldwide. PCV2 infection is associated with various diseases, including porcine circovirus-associated disease (PCVAD), postweaning multi-systemic wasting syndrome (PMWS), porcine dermatitis and nephropathy syndrome (PDNS), and reproductive failure in pigs, especially when infected with porcine reproductive respiratory syndrome (PRRS) virus, porcine parvovirus (PPV), and mycoplasma, which lead to decreased production efficiency.

PCV2 is a small, non-enveloped virus with a circular, single-stranded DNA genome, and it is comprised of two major proteins, ORF1 and ORF2. The ORF1 on the positive strand encodes two nonstructural proteins (Rep-Rep’), which are essential for viral replication. The OFR2, namely capsid protein on the complementary strand, binds to the host receptor and induces immune responses [1]. Based on ORF2 nucleotide sequences, the genotypes of PCV2 have been commonly classified as PCV2a, 2b, 2c, 2d, and 2e [2–5].

PCV2a was the dominant genotype in pig herds until the early 2000s. After introduction of the PCV2a-derived vaccine in 2006, PCV2b became the dominant genotype inthe late 2000s [6–8]. Since the PCV2d genotype was first reported in China and the US in the early of 2010s, the rapid spread of PCV2d has been documented worldwide [4, 9]. In 2013, the PCV2d genotype was first isolated from cases of vaccine failure in Korea [10] and appeared highly prevalent afterward [11]. Besides, the PCV2d genotype was reported to have virulence comparable to PCV2a and 2b [12].

Commercial vaccines based on PCV2a have been applied to the field since 2006 and are recognized as an effective tool to control outbreaks of PCVAD. However, many outbreaks of PCVAD have been recently reported from the farms vaccinated with PCV2a-based vaccines, from which field viruses of the PCV2d genotype have been isolated along with disappearance of PCV2a genotype strains. The genotype shift in the field has suggested that PCV field isolates may have gone through antigenic mutations, leading to escape from immune responses induced by commercially available vaccine strains [13–15]. Due to rapid change of PCV2 genotype and continuous reports of vaccine failure in the field, it is necessary to investigate the prevalence of PCV2 infection and genetic variation. Therefore, we isolated PCV2 field viruses from swine farms vaccinated mostly with commercial PCV2a-based vaccines from 2016 to 2018, and investigated genetic diversity and difference of cross-neutralization activity of the field viruses.

## Results

### Genetic analysis of PCV2 circulating in swine farms of Korea

**Fig. 1:**
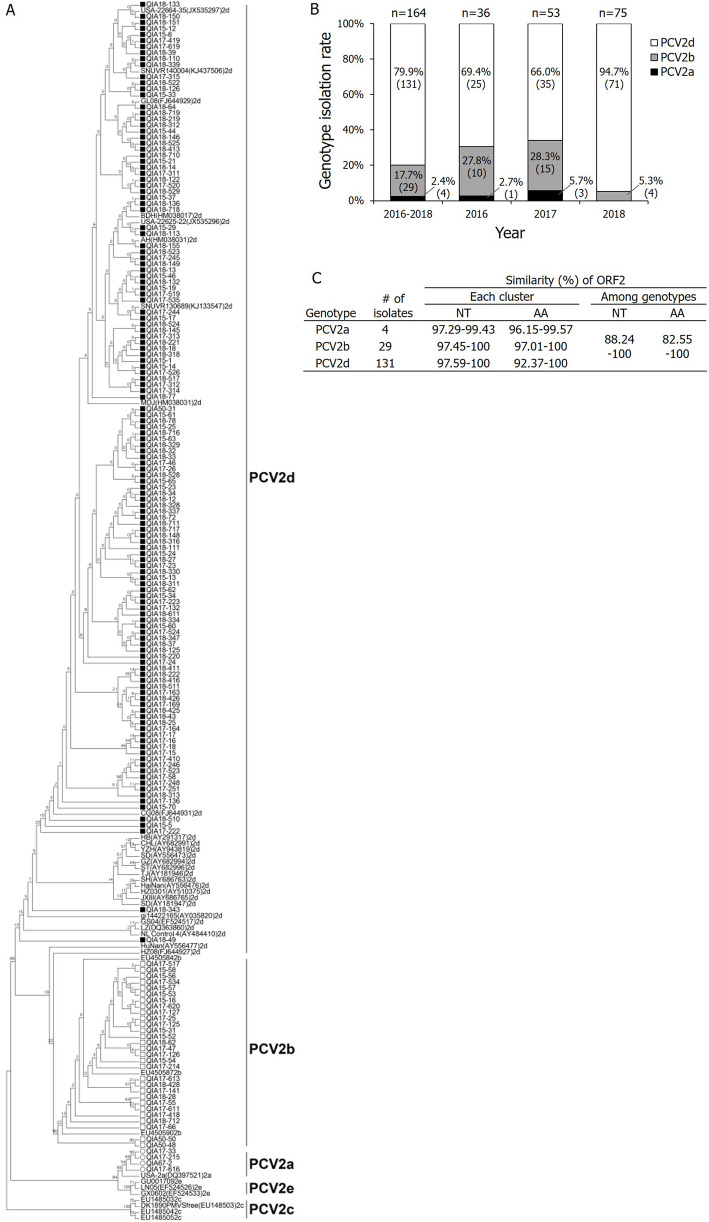
Genetic analysis of PCV2 ORF2 isolated from 2016 to 2018 in Korea. ORF2 sequences of 164 PCV2 isolates were analyzed with 47 PCV2 references. **a** Phylogenetic-tree analysis of Korean isolates by genotypes, PCV2a (○), PCV2b (□), and PCV2d (■). The phylogenetic tree was constructed using MEGA6 software with the neighbor-joining method, and bootstrap values were calculated on 1000 replicates. **b** Genotype isolation rate for PCV2 field viruses according to isolation year. The parentheses indicate the number of isolates. **c** ORF2 nucleotide (NT) and amino acid (AA) sequence similarity of PCV2 isolates

In ORF2 PCR of the clinical samples, PCV2-positive ratio of tissues (50.3%) were higher than that of sera (24.4%) in 2016. In the following detection, the PCV2 -positive ratio of tissues were 69.5 and 52.4% for 2017 and 2018, respectively. Totally, 57.5% (288/501) of the tissues were positive (Table 1). Regarding the phylogenetic analysis based on ORF2 sequence (*n* = 164), all of the Korean isolates were classified into PCV2a (*n* = 4), 2b (*n* = 29), and 2d (*n* = 131), with 2.4, 17.7, and 79.9% infection rates, respectively (Fig. 1a and b). The isolation rate of PCV2a, 2b, and 2d field viruses from 2016 to 2017 was 2.7 to 5.7%, 27.8 to 28.3%, and 69.4 to 66.0%, respectively. In 2018, PCV2a was not detected, and PCV2b was isolated as 5.3% in the field, whereas PCV2d genotypes accounted for 94.7% of field isolates. In the pair-wise comparison of ORF2 NT and AA sequences, the NT (AA) sequence similarity of PCV2a, PCV2b, and PCV2d was 97.3 to 99.4% (96.2–99.6%), 97.5 to 100% (97.0–100%), and 97.6 to 100% (92.4–100%), respectively, whereas the NT (AA) sequence similarity of all genotypes was 88.2 to 100% (82.6–100%) (Fig. 1c).

**Table 1:** Information of clinical samples

	No. of farms	
	Tissues	Sera
2016	157 (50.3)	361 (24.4)
2017	178 (69.5)	–
2018	166 (52.4)	–
Total	501 (57.5)	361 (24.4)

Parentheses is the PCV2-positive ratio (%). Multiple clinical samples collected from single farm were counted as one

### Diverse reactivity of PCV2-specific mAbs against PCV2 genotypes

**Fig. 2:**
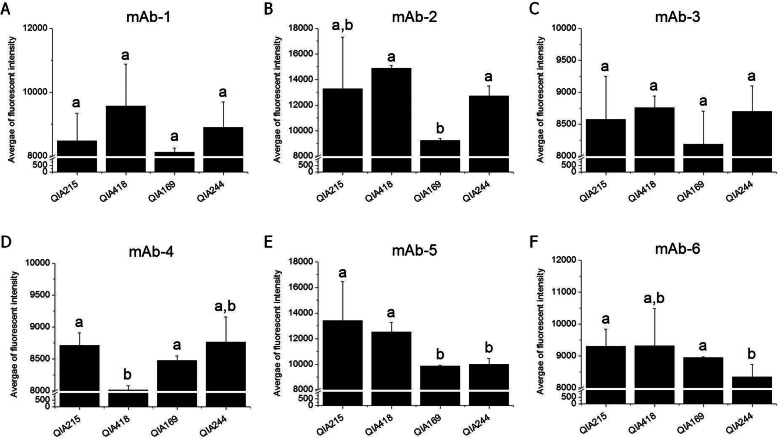
Diverse antigenic reactivity of mAbs to PCV2 genotypes. 200 TCID_50_ of QIA215, QIA418, and two PCV2d isolates (QIA169 and QIA244) were infected to PK15 cells, and the infected cells were reacted with mAb-1 (**A**), mAb-2 (**B**), mAb-3 (**C**), mAb-4 (**D**), mAb-5 (**E**), and mAb-6 (**F**). The average of fluorescent intensity per 1 × 10^4^ cellular nuclei was measured by ArrayScan VTI HCS. Different superscript letters indicate significant differences (*p* < 0.05)

**Fig. 3:**
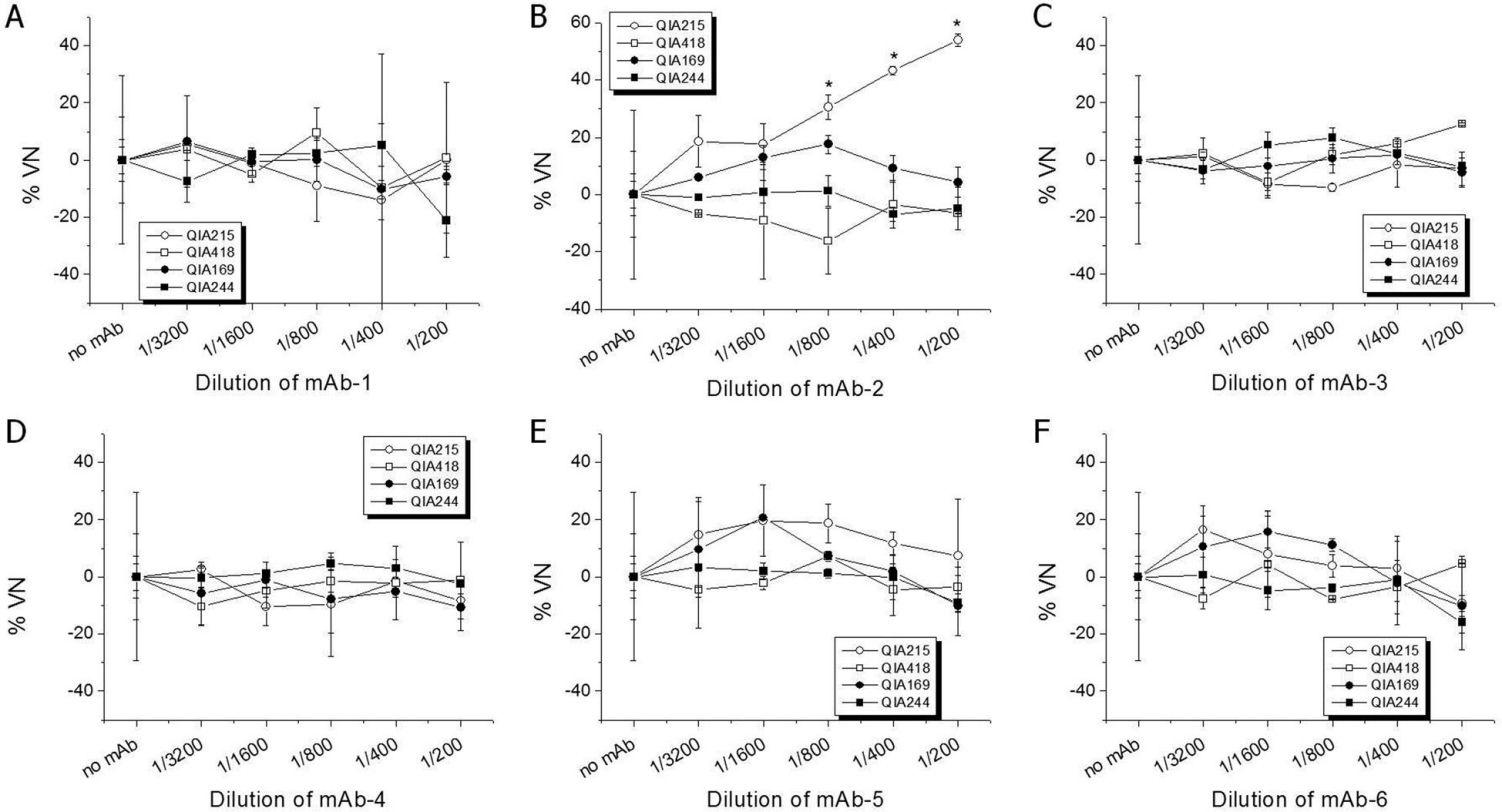
Neutralization assay using mAbs for PCV2 genotypes. 200 TCID_50_ of QIA215 (○), QIA418 (□), QIA169 (●), and QIA244 (■) were neutralized withdiluents (1/200 to 1/3200) of mAb-1 (**a**), mAb-2 (**b**), mAb-3 (**c**), mAb-4 (**d**), mAb-5 (**e**), and mAb-6 (**f**), and the neutralized mixtures were infected on PK15 cells. The fluorescent intensity of PCV2-positve cells at day 5 of post-infection was determined as a %VN by immunostaining with mAb-2 having the reactivity against all PCV2 genotypes. *Significantly different from control at *p* < 0.05

Based on the ORF2 AA sequence, the representative strains (QIA215, QIA418, QIA169, and QIA244) were examined for antigen-binding activity to mAbs. The six PCV2-specific mAbs showed diverse reactivity to PCV2 field viruses. As shown in Fig. 2, mAb-1 and mAb-3 had similar reactivity to PCV2 field viruses. In the case of mAb-2, it had significantly lower reactivity to QIA169 than the others. mAb-4 had significantly lower reactivity to QIA418 than other viruses. mAb-5 had significantly higher reactivity to QIA215 and QIA418 than other viruses. mAb-6 showed significantly lower reactivity to QIA244 than other viruses. The mAbs (1/200–1/3200 dilution) were mixed with the representative strains to evaluate neutralization activity. In results (Fig. 3), most of the mAbs did not have neutralization activity against all of field viruses, whereas only mAb-2 showed neutralization activity (%VN of 30.5–53.9%) only against QIA215 at 1/200 to 1/800 dilution.

### Cross-virus neutralization of anti-PCV2 sera

**Fig. 4:**
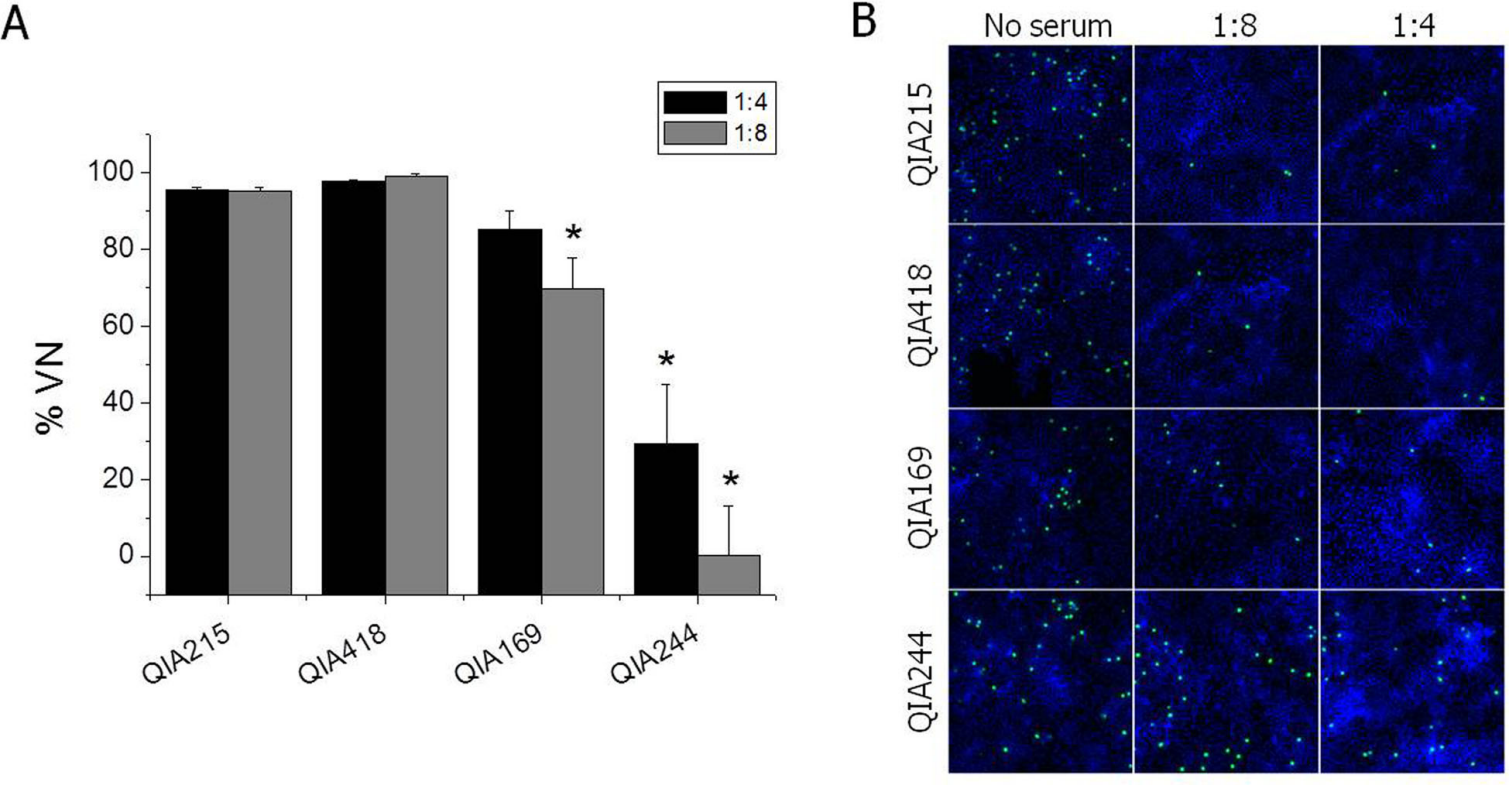
Viral neutralization assay using anti-PCV2 pig sera. **a** Anti-sera collected from SPF pigs vaccinated with Ingelvac CircoFLEX were neutralized with homologous and heterologous genotypes of PCV2. *Significantly different from control at *p* < 0.05. **b** The %VN of Fig. 4a was converted from the number of PCV2-positive cells counted per 1 × 10^4^ cells using ArrayScan VTI HCS. Nuclei were counterstained with Heches33258. The merged images were magnified as 100 ×

**Fig. 5:**
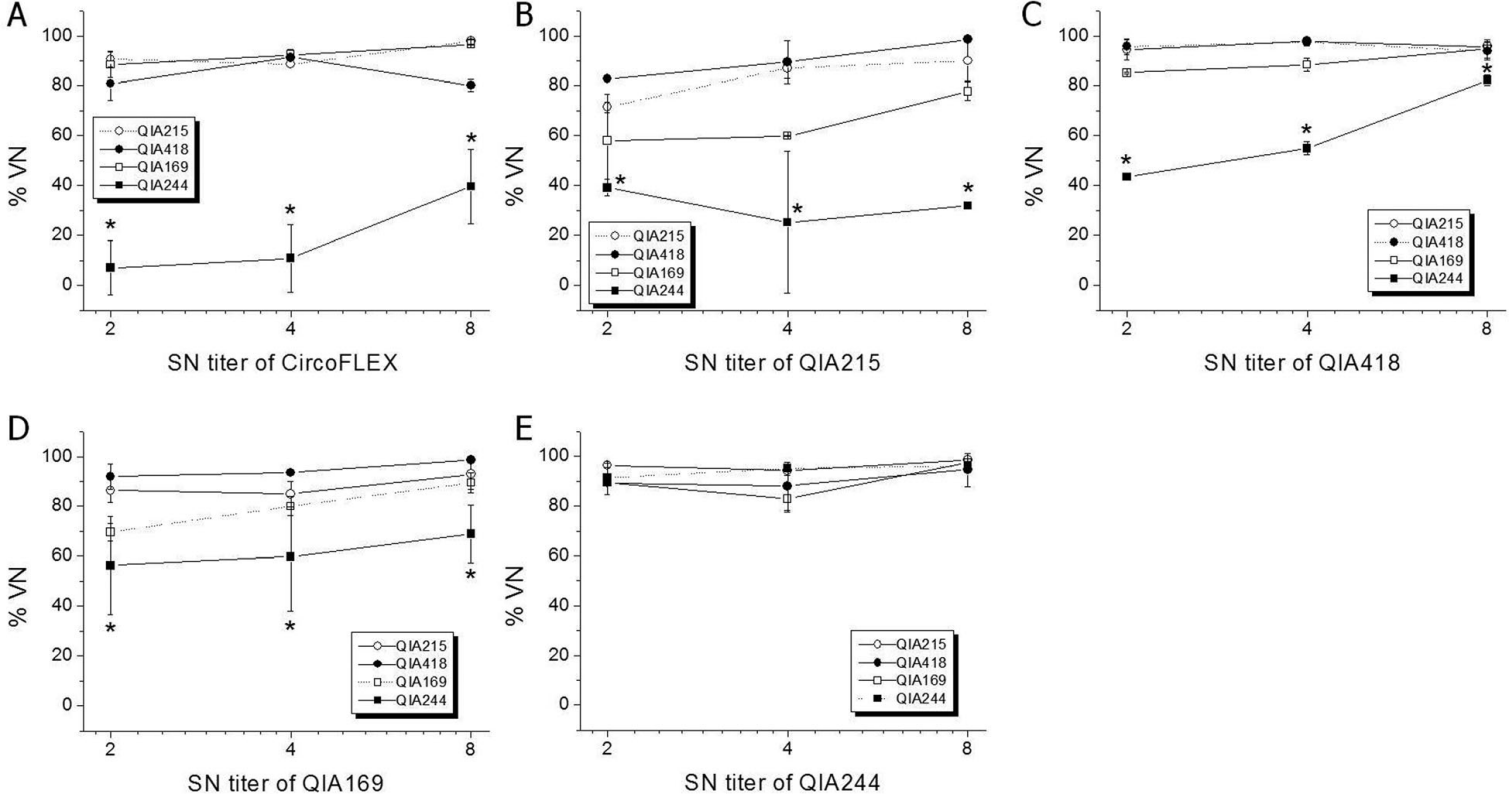
Evaluation of cross-protection capability (%VN) among different PCV2 genotypes. The anti-sera collected from guinea pigs vaccinated with Ingelvac CircoFLEX (**a**), QIA215 (**b**), QIA418 (**c**), QIA169 (**d**), and QIA244 (**e**) were neutralized with four PCV2 viruses: QIA215 (○), QIA418 (□), QIA169 (●), and QIA244 (■). Cross-protection efficiency among genotypes was evaluated using anti-sera of 1:2 to 1:8 SN titer against homologous viruses (dot line). *Significantly different from control at *p* < 0.05

**Fig. 6:**
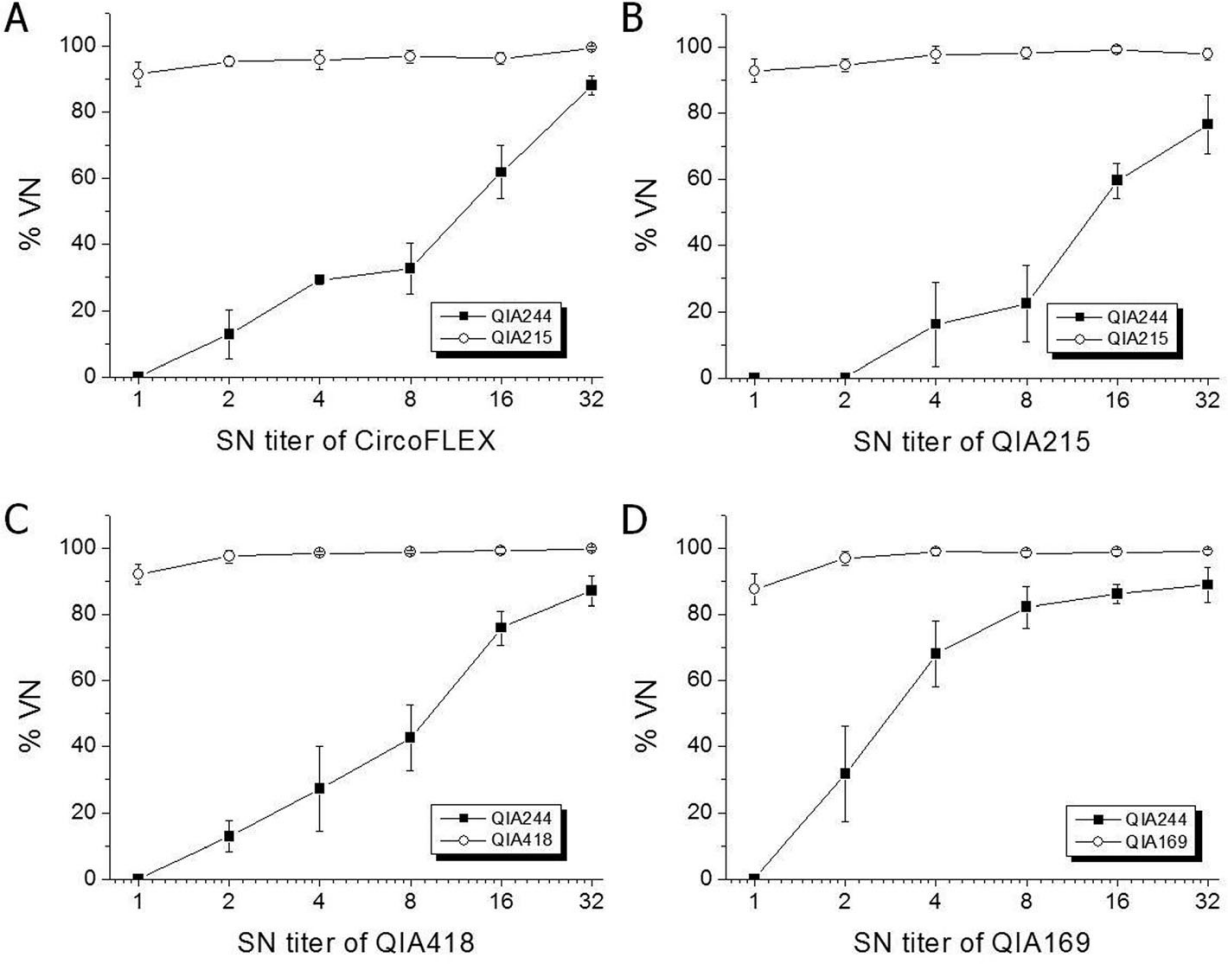
Further evaluation of viral neutralization activity (%VN) against QIA244 using anti-PCV2 guinea pig sera derived from different genotypes of PCV2. QIA244 isolates were neutralized with anti-sera of Ingelvac CircoFLEX (**a**), QIA215 (**b**), QIA418 (**c**), and QIA169 (**d**) of up to 1:32 SN titer. As a control, homologous viruses were compared for each anti-serum. *Significantly different from control at *p* < 0.05

To evaluate cross-neutralization activity among PCV2 strains, anti-PCV2 sera produced from pigs and guinea pigs were reacted with homologous and/or heterologous (genotype) viruses. For anti-pig sera injected with a commercial vaccine containing PCV2a capsid protein, the %VN was larger than 95% against QIA215 and QIA418 at 1:4 and 1:8 SN titer, and was significantly lower against PCV2d strains (69.9% for QIA169 at 1:8 SN titer, 29.3 and 0.3% for QIA244 at 1:4 and 1:8 SN titer, respectively) than against QIA215 or QIA418 (Fig. 4). When anti-guinea pig sera were applied to the virus neutralization assay, the %VN of anti-commercial vaccine sera was 80 to 95% against QIA215, QIA418, and QIA169 at all SN titers (Fig. 5a). The %VN of anti-QIA215 sera was approximately 80 to 100% against the homologous viruses, QIA418 and QIA169, without statistical difference at all SN titers (Fig. 5b). The %VN of anti-QIA418 sera was more than 85% against the homologous viruses, QIA215 and QIA169, at all SN titers (Fig. 5c). The %VN of anti-QIA169 sera was 80 to 100% against the homologous viruses, QIA418 and QIA215, without statistical difference (Fig. 5d). It was noted that the %VN of all of anti-sera except anti-QIA244 sera was statistically lower against QIA244 than the homologous or other heterologous viruses. The %VN against the QIA244 strain was 7.0 to 39.6%, 25.2 to 39.1%, 43.5 to 82.3%, and 56.4 to 68.8% for anti-sera of CircoFLEX, QIA215, QIA418, and QIA169 at all SN titers, respectively. Moreover, the %VN of anti-QIA244 sera was 80 to 98% against all of the homologous and heterologous viruses at all SN titers without statistical difference (Fig. 5e). In the further evaluation of viral neutralization activity against QIA244 using anti-PCV2 guinea pig sera between < 1:2 and 1:32 SN titer, the %VN against QIA244 were 0 to 88.0%, 0 to 76.6%, 0 to 87.0%, and 0 to 89.0% at all SN titers for anti-sera of CircoFLEX, QIA215, QIA418, and QIA169, respectively, which were significantly lower than those against each homologous virus (Fig. 6).

## Discussion

PCV2a was the only genotype isolated in the field before the PCV2a-based vaccination. However, since the PCV2a vaccine was applied to the field in 2006, the genotype shift to PCV2b and PCV2d emerged and was expedited over time, and PCV2d became the dominant genotype around the world. In the present study, 164 PCV2 field viruses were isolated from clinical samples from 2016 to 2018 in Korea to investigate genetic diversity and antigenic relatedness based on PCV2-specific mAb reaction and cross-neutralization activity of anti-PCV2 sera. ORF2 (capsid protein) sequence analysis showed that PCV2d has become a predominant genotype circulating in Korean swine farms with approximately 80% of isolation rates, whereas other genotypes were hardly isolated, with less than 3 and 18% for PCV2a and PCV2b, respectively. Further, the Korean field isolates of PCV2d showed up to 82.6% with PCV2a isolates, and also ranged from 92.4 to 100% among the same genotype for amino acid sequence similarity of the capsid protein. The prevalence of PCV2 genotypes was similar to the recent study [16]. Even though PCV2a was hardly found, there has been continuous isolation of PCV2b with even a small proportion in Korean swine farms. However, it was anticipated that the genotype may completely disappear in near future, because PCV2a-based commercialized vaccine neutralized PCV2b isolate better than PCV2d isolate as shown in this study (Fig. 4), and was highly protective against PCV2b isolates in pigs [17]. The possible effect of the PCV2a vaccination on the genotype shift was documented in previous reports [13–15], suggesting that the vaccination has the great potential to be a cause of vaccine-escaping mutants in the field. Therefore, along with the drastic genotype shift, the wide range of amino acid sequence differencesin capsid protein for Korean recent field isolates suggested that the substantial antigenic variability including neutralization activity of the viruses also should be investigated. Despite extensive documentation of the genetic variation of PCV2, the antigenic diversity of PCV2 has not been well studied. The antigenic diversity among PCV2 strains was first demonstrated by the use of monoclonal antibodies [18], whereas there was no difference of antigenic reactivity in Korean field PCV2d isolated from a vaccine failure farm [10]. We also investigated the antigenic diversity among PCV2 field viruses by the antigenic reaction and virus neutralizing activity of mAbs and anti-PCV2 sera. As shown in Fig. 2, mAb-5 showed strong reaction with PCV2a, and 2b, but not PCV2d genotype. Two mAb (mAb-1 and mAb-3) were bound to all of the genotypes in similar specificity. The others (mAb-2, mAb-4, and mAb-6) were reacted with PCV2 viruses, regardless of genotype. Also, it was noted that mAb-2 had neutralizing activity only against QIA215, as shown in Fig. 3. The previous study also reported that mAb 8E4, an antibody produced against a PCV2a isolate, neutralized only PCV2a [19]. Even if there has been no information about neutralization activity between the mAbs and PCV2d strains, the diverse reactivity of the mAbs against field isolates in this study suggested that the antigenic variability of neutralizing or non-neutralizing epitopes may exist among PCV2 genotypes as well as within the same genotype, failing to find genotype-specific mAb, as in the previous studies.

The neutralization activity of anti-PCV2 sera varied among the field viruses. Importantly, anti-PCV2 swine sera of the current commercial vaccine had low virus neutralization activity by less than 30% VN against a PCV2d strain, QIA244. At the same time, in cross-neutralization using anti-PCV2 guinea pig sera, each of the anti-PCV2 sera effectively neutralized the same genotype virus or the different genotype viruses except one of the PCV2d strains, QIA244. As shown in the results, while a PCV2d virus, QIA169, was efficiently neutralized by anti-sera of all viruses, the other PCV2d virus, QIA244, was not fully neutralized by anti-guinea pig sera of up to 1:32 SN titer. The low neutralization activity against the PCV2d virus, QIA244, was expected from the lowest ORF2 amino acid sequence similarity of QIA244 with other viruses (91.6% between QIA215 and QIA418; 89.7% between QIA215 and QIA169; 89.4% between QIA215 and QIA244; 93.1% between QIA418 and QIA169; 92.8% between QIA418 and QIA244; and 99.3% between QIA169 and QIA244). Importantly, the low neutralization activity within the PCV2d viruses (QIA244 vs. QIA169) was unexpected. In terms of cross-protection among different genotype viruses, experimental studies for cross-protection between genotypes showed that PCV2a- or PCV2b-based commercial vaccine was protective against concurrent infection of different genotype viruses (PCV2b and PCV2d strains) in pigs [20]. Nevertheless, experimental evidence from the controlled studies also supported that a viremia was effectively decreased by homologous vaccine more than heterologous vaccine in a concurrent PCV2a and PCV2d challenge [21]. Besides, viremia and shedding of virus were still observed in PCV2a-vaccinated pigs, although the viral load was decreased after the PCV2d challenge [22], which was observed from the studies using challenging viruses of other genotypes [12, 22–24]. Moreover, mutant PCV2b (PCV2d) was isolated from vaccinated farms [4, 10]. Therefore, the different virus neutralization efficiency among the same genotypes in this study and inconsistent protective efficacy of vaccine against the same genotype challenges in the previous studies suggest that the neutralization activity of PCV2 may be highly diverse among field strains in the field. Besides, since we observed differences of amino acid residues of the capsid protein between QIA169 and QIA244, further studies to clarify which amino acid is responsible for the difference of neutralization activity are needed to be conducted by mutating the amino acid residue of QIA244 to that of QIA169.

## Conclusions

This study confirmed genetic variation including a dramatic genotype shift (from PCV2a or PCV2b to PCV2d) and demonstrated different virus neutralization activity of field isolates recently circulating in Korean swine farms, suggesting that the strain-based difference in virus neutralization capability should be considered for more effective control by vaccination.

## Methods

### Clinical samples and isolation of viruses

Clinical samples including 361 sera and 501 tissues (lungs and lymph nodes) were obtained from the nationwide farms (*n* = 862) where the disease diagnosis was requested due to the problem of respiratory and wasting syndromes from 2016 to 2018. Tissues and sera were collected in 2016, and only tissues were collected from 2017 to 2018 due to the higher viral detection efficiency in tissues. We described the detailed sample collection in Table 1. All PCV2-positive clinical tissue sampleswere used for viral isolation and genetic analysis.

The tissue samples (300–500 mg) were homogenized in phosphate-buffered saline (PBS) with MagNALyser Green Beads (Roche Diagnostics, Mannheim, Germany) and Precellys 24 homogenizer (Bertin Technologies, Montigny-le-Bretonneux, France) according to the manufacturer’s instructions. Homogenized tissues were centrifuged at 13,000 rpm, at 4 °C for 5 min, and the supernatants were transferred into sterile Eppendorf tubes. The tissue homogenates and sera were kept at − 70 °C until use. To detect PCV2 in the clinical samples, the ORF2 gene (493 bp) was amplified using the HotStarTaq Plus Master Mix Kit and ORF2-specific forward primers (5′-CACGGATATTGTAGTCCTGGTCG-3′) and reverse primers (5′-CGCACCTTCGGATATACTG-3′).

PCV2-positive clinical tissue extracts were infected with PK15 cells, which were all types of PCV-free as confirmed by PCR, and PCV2 isolates including PCV2a, 2b, and 2d genotypes were propagated with high titer (10^5^–10^6^ TCID_50_/mL). Viral titration was conducted following the previous report [25] and viral titers were determined by immunostaining using PCV2-specific monoclonal antibodies (mAbs). Based on the amino acid sequence of ORF2 and growth efficiency in PK15 cells, representative isolates of two PCV2d (QIA169 and QIA244) were selected for evaluation of cross-reactivity along with PCV2a (QIA215) and PCV2b (QIA418) genotypes.

### Preparation of monoclonal antibodies (mAbs) and anti-sera

Six PCV2-specific mAbs named mAb-1, mAb-2, mAb-3, mAb-4, mAb-5, and mAb-6 were kindly provided by Median Diagnostics Inc. and BioPOA Ltd., Korea. These mAbs were produced by immunizing mouse (Balb/c, *n* = 3) with PCV2a viruses and selected based on the specific reactivity tocapsid proteins of the PCV2a viruses. Anti-PCV2 sera of specific pathogen-free (SPF) pigs vaccinated with Ingelvac CircoFLEX (Boehringer Ingelheim Vetmedica, Rohrdorf, Germany) were also kindly provided by Median Diagnostics Inc. The anti-PCV2 pig sera were pooled to be used for viral neutralization assay. Anti-PCV2 sera of guinea pigs were prepared by injection of QIA215, QIA418, QIA169, QIA244, and Ingelvac CircoFLEX into guinea pigs (three heads for each virus). For the first injection, the field viruses (10^6^ TCID_50_/mL) were mixed with Freund’s complete adjuvants and intramuscularly injected into the guinea pigs. After 2 weeks, the second injection was conducted with the field viruses (10^6^ TCID_50_/mL) mixed with Freund’s incomplete adjuvants. The commercial vaccine was injected following the same immunization schedule as the field viruses. After 2 weeks of the second injection, all guinea pigs were euthanized, and the sera were collected. The sera were aliquoted and stored at − 20 °C until use. All guinea pigs were euthanized by an intramuscular injection of ketamine/xylazine under the guideline for use of controlled drugs.

### Nucleotide (NT)/amino acid (AA) sequence and phylogenetic analysis

Viral genomic DNAs were extracted from tissue homogenates and sera using the DNeasy Blood &Tissue Kit (Qiagen, Hilden, Germany) according to the manufacturer’s instructions. The full-length of the ORF2 gene was amplified using the HotStarTaq Plus Master Mix Kit and ORF2-specific forward primers (5′-GGAATGGTACTCCTCAACTG-3′) and reverse primers (5′-CTCGTCTTCGGAAGGATTAT-3′). The resulting polymerase chain reaction (PCR) products (1061 bp) were purified by the QIAquick PCR Purification Kit (Qiagen) and were confirmed by DNA nucleotide sequencing (Macrogen, Seoul, South Korea).

To analyze the genetic relatedness between the PCV2 isolates detected from swine farms in Korea, we performed multiple sequence alignment with CLC Main Workbench (Qiagen, Version 7.0.3). A neighbor-joining tree was constructed using MEGA software (Version 7.0), and the reliability of the constructed tree was evaluated by bootstrap analysis of 1000 replications.

### Immunofluorescence assay

PK15 cells were infected with the same titer of PCV2a, 2b, 2d-1 and 2d-2 as 200 TCID_50_. For evaluating the binding reactivity of PCV2 and mAbs, infected PK15 cells were fixed with 80% acetone for 10 min at − 20 °C. After washing with 1 × PBS, mAbs (1:200) as primary antibodies were reacted by incubating for 1 h at room temperature (RT). After rinsing, Alexa fluor™ 488 goat anti-mouse immunoglobulin G (IgG, 1:200, Invitrogen, CA, USA) as a secondary antibody was incubated for 30 min at RT. The nuclei were stained for 5 min with Hoechst33258 (Invitrogen) diluted in 1 × PBS (1:10,000). For the detection of PCV2-infected cells, fluorescent intensity of PCV2-positive cells per 1 × 10^4^ cellular nuclei was measured by ArrayScan VTI HCS (Thermo Scientific, MA, USA), and the number of PCV2-positive cells was counted with by the naked eye.

### Viral neutralization assay

Viral neutralization assay was conducted following the method of Meerts and colleagues [26]. Briefly, 200 TCID_50_ PCV2 at a volume of 100 μL was incubated for 1 h at 37 °C with 100 μL of serially diluted mAbs or anti-PCV2 sera. After incubation, this mixture was added to 5 × 10^3^ PK15 cells in four wells of a 96-well plate. After 2 h at 37 °C, the cell culture media were washed twice in 1 × PBS, and fresh medium was added. Cells were fixed 5 days later. PCV2-infected PK15 cells were stained aspreviously described. The neutralization activity of mAbs and serum was expressed as a percentage of the viral neutralization (%VN) using the number of PCV2-positive cells.

$$ \% VN=100-\left(\frac{\mathrm{the}\ \mathrm{number}\ \mathrm{of}\ \mathrm{PCV}2\ \mathrm{positive}\ \mathrm{cells}\ \mathrm{with}\ \mathrm{serum}\ \left(\mathrm{mAb}\right)}{\mathrm{the}\ \mathrm{number}\ \mathrm{of}\ \mathrm{PCV}2\ \mathrm{positive}\ \mathrm{cells}\ \mathrm{with}\mathrm{out}\ \mathrm{serum}\ \left(\mathrm{mAb}\right)}\times 100\right) $$

### Statistical analysis

The results were expressed as the mean ± standard error (SE) for triplicate experiments (*n* = 3). The statistical significance was determined using Statistica5.5 (StatSoft, OK, USA) with one-way analysis of variance (ANOVA) and post-hoc comparisons between the control group and each treatment group using Duncan’s multiple comparison test. A *p* value < 0.05 was considered to be statistically significant.

## Data Availability

All data generated or analyzed during this study are included in this published article. The phylogenetic data are available in the TreeBase at http://purl.org/phylo/treebase/phylows/study/TB2:S26750?x-access-code=32ca0fc83c3df17e3841fb5c80fae063&format=html. The study ID on TreeBase is S26750.

## Abbreviations

AA: Amino Acid
mAb: monoclonal antibody
NT: Nucleotide
ORF: Open Reading Frame
PCV: Porcine circovirus
PCVAD: Porcine circovirus-associated disease
PDNS: Porcine dermatitis and nephropathy syndrome
PMWS: Postweaning multi-systemic wasting syndrome
PPV: Porcine parvovirus
PRRS: Porcine reproductive respiratory syndrome
SN: Serum neutralization
TCID: Tissue culture infective dose
VN: Viral neutralization
